# ChatMDV: reducing technical barriers in bioinformatics analysis using large language models

**DOI:** 10.1093/gigascience/giag073

**Published:** 2026-06-19

**Authors:** Maria Kiourlappou, Peter Todd, Yaxuan Kong, Jayesh Hire, Sibgathullah Furquan Nawab Mohammed, Devika Agarwal, Martin Sergeant, Stefan Zohren, Brian Marsden, Jim Hughes, Stephen Taylor

**Affiliations:** Centre for Human Genetics, Nuffield Department of Medicine, University of Oxford, Oxford, OX3 7BN, UK; Centre for Human Genetics, Nuffield Department of Medicine, University of Oxford, Oxford, OX3 7BN, UK; Department of Engineering Science, University of Oxford, Oxford, OX1 3PJ, UK; Centre for Human Genetics, Nuffield Department of Medicine, University of Oxford, Oxford, OX3 7BN, UK; Centre for Human Genetics, Nuffield Department of Medicine, University of Oxford, Oxford, OX3 7BN, UK; Kennedy Institute of Rheumatology, Nuffield Department of Orthopaedics, Rheumatology, and Musculoskeletal Science, University of Oxford, Oxford, OX3 7FY, UK; Weatherall Institute of Molecular Medicine, Radcliffe Department of Medicine, University of Oxford, Oxford, OX3 9DS, UK; Department of Engineering Science, University of Oxford, Oxford, OX1 3PJ, UK; Centre for Human Genetics, Nuffield Department of Medicine, University of Oxford, Oxford, OX3 7BN, UK; Centre for Medicines Discovery, Nuffield Department of Medicine, University of Oxford, Oxford, OX3 7FZ, UK; Weatherall Institute of Molecular Medicine, Radcliffe Department of Medicine, University of Oxford, Oxford, OX3 9DS, UK; Centre for Human Genetics, Nuffield Department of Medicine, University of Oxford, Oxford, OX3 7BN, UK

**Keywords:** natural language interfaces, data visualisation, bioinformatics, large language models, FAIR principles

## Abstract

**Background:**

The rapid advancement in single-cell, spatial omics, imaging, and genomic technologies requires robust analytical and visualisation platforms capable of managing complex biological data. Tools such as Multi-Dimensional Viewer (MDV) offer comprehensive interfaces for data exploration but often require advanced computational expertise and manual configuration to generate visualisation outputs, limiting accessibility for many users.

**Results:**

We present ChatMDV, a natural language interface integrated with MDV that enables users to generate high-quality, interactive visualisations and analyses through natural language commands. ChatMDV employs a retrieval-augmented generation pipeline in combination with large language models to translate user queries into executable, reproducible Python code and interactive output. This conversational layer facilitates both exploratory and targeted analyses in diverse biological domains. We demonstrate ChatMDV’s capabilities using 3 datasets of increasing complexity: the Peripheral Blood Mononuclear Cells 3K single-cell RNA-sequencing (scRNA-seq) dataset, the lung cancer atlas scRNA-seq dataset included in the Human Cell Atlas, and the longitudinal TAURUS study scRNA-seq dataset. Across all use cases, ChatMDV produced high-quality, reproducible visualisations from simple natural language queries, achieving a high semantic success rate between 79% and 97% when visualising the datasets.

**Conclusions:**

By bridging the gap between natural language processing and bioinformatics visualisation, ChatMDV reduces technical barriers, enhances reproducibility, and supports more inclusive scientific inquiry. Its modular design and adherence to Findability, Accessibility, Interoperability, and Reuse (FAIR) principles make it a scalable and adaptable framework for accelerating biological data analysis.

## Key Points

- ChatMDV enables users to create interactive visualisations from biological datasets using natural language.
- The system combines large language models with Multi-Dimensional Viewer’s graphical platform to simplify data exploration.
- It supports reproducibility, adaptability, and Findability, Accessibility, Interoperability, and Reuse (FAIR) data practices, making it suitable for a wide range of users and use cases.

## Background

The increasing scale and complexity of high-throughput biological data, particularly from single-cell, spatial, and imaging omics, has created a critical need for accessible and scalable visualisation tools. Recent advances in imaging and sequencing technologies have allowed researchers to interrogate biological systems with unprecedented resolution.

Although platforms such as Vitessce [1], Napari [2], and Multi-Dimensional Viewer (MDV) [3] have significantly advanced interactive exploration of multimodal data, many of these tools require manual configuration or programming knowledge, creating barriers for researchers without computational training. In particular, Vitessce [1] provides web-based linked views for spatial and single-cell datasets via a graphical interface designed to support interactive data exploration; however, its customisation and layout definitions require manual editing of JSON configuration files, limiting flexibility for nontechnical users. Napari [2] excels in n-dimensional microscopy data visualisation, offering a Python-based desktop interface for image segmentation, annotation, and plug-in–based analysis. While it is extensible and performant, it is primarily optimized for image data rather than tabular or multimodal omics data. MDV [3] is a web-based interactive analytics and visualisation tool that allows a wide variety of complex datasets to be viewed, annotated, and shared. For example, MDV allows access and integration of data from multiomic single-cell, spatial imaging, and genomic projects via its user-friendly graphical interface. While MDV streamlines many aspects of data visualisation, tasks that require repetitive actions across multiple features (e.g., constructing charts for visualising gene expression for many genes across a disease condition) may introduce interaction overhead. Such operations, where multiple charts are required to understand the data, are often laborious for the user. Furthermore, when users are presented with such large datasets for the first time, they may face challenges in identifying appropriate starting points for analysis, particularly when navigating high-dimensional datasets with numerous variables and potential visualisation options. In parallel, specialised toolkits such as CAT Bridge support integrative multiomics analysis workflows [4], while frameworks such as SeuratExtend aim to streamline single-cell RNA-seq (scRNA-seq) analysis through integrated and user-friendly pipelines [5].

Graphical user interfaces (GUIs), although designed to promote ease of use, accessibility, and immediate visual feedback, can get overcomplicated, be difficult to integrate with other preprocessing and analysis pipelines, and offer limited support for automation. Meanwhile, command-line interfaces (CLIs) might enable efficient automation of repetitive tasks and allow greater flexibility for pipeline customisation and integration but often require a steep learning curve, and they are more error prone as small syntax errors may cause commands to fail and alienate nonprogrammers. This usability gap continues to slow analysis workflows, limit reproducibility, and hinder broader data interpretation. In addition, first-time users may find the GUI overwhelming when navigating large and complex datasets, particularly in identifying appropriate entry points for analysis or formulating initial queries.

To address this gap, natural language–based interfaces have emerged as a promising solution for data exploration, driven by advances in natural language processing (NLP) and large language models (LLMs) [6–8]. Open-source Python frameworks such as LangChain [9] have been developed to facilitate the creation of robust, modular LLM-powered applications. Selected functionalities offered by the LangChain framework encompass agentic systems capable of operating at varying degrees of autonomy [10]. These systems facilitate complex decision-making processes by dynamically invoking external tools. The usefulness of agentic systems has been demonstrated by their application in tools such as FlowAgent [11] and CellAgent [12], allowing agent-based automation for scRNA-seq data analysis. Recent work has also evaluated the use of LLMs such as ChatGPT [13] for scientific workflow development, highlighting both their potential and limitations when applied to structured analytical tasks [14]. In addition, the emergence of foundation models for genomics further demonstrates the expanding role of artificial intelligence (AI)–driven approaches in biological data analysis [15]. In addition, LangChain [9] supports retrieval-augmented generation (RAG) [16], a paradigm that enhances the output of LLMs by incorporating pertinent information retrieved from external knowledge sources directly into the model’s input context. This integration yields responses that are more accurate, contextually grounded, and aligned with the information landscape relevant to the task.

By leveraging NLP and LLMs, we have developed **ChatMDV**, a natural language interface for MDV that effectively addresses these challenges. ChatMDV enables users to generate, edit, and explore interactive biological visualisations using plain language prompts, without needing to write code. When a natural language prompt is submitted to ChatMDV through the interactive chatbot interface, ChatMDV generates the relevant Python code that is executed on the server, generating a new MDV view containing 1 or more chart(s) addressing the question. For example, a user can direct ChatMDV to “Plot a chart of the UMAP components,” and ChatMDV will automatically identify the corresponding columns within the dataset and generate a suitable scatterplot (Supplementary Video 1). Users can then ask follow-up questions to modify the visualisation (Supplementary Video 2) or create multiple plots within the same view, for example, “Can you plot the 20 most important genes in the dataset and show their expression in box plots against the lung tissue?” (Supplementary Video 3). ChatMDV can filter and subset datasets through natural language queries such as “Compare inflammation severity scores across patients filtered by disease and remission status,” adding an interactive selection dialog plot for data filtering (Supplementary Video 4). It can also generate a text box output to display dataset summaries or key statistics (Supplementary Videos 5 and 6). When working with scRNA-seq data stored in Anndata [17] objects, ChatMDV can integrate cell and gene matrices by linking predefined gene identifiers, accurately mapping between gene names and gene IDs (Supplementary Videos 7 and 8). By combining its Python execution environment with the reasoning capabilities of the underlying LLM, ChatMDV can leverage its learned knowledge of scRNA-seq analysis workflows and generate code using established packages such as Scanpy [18] to perform differential gene expression analysis and visualise it using suitable plots (Supplementary Video 9). ChatMDV further assists users by formulating biologically meaningful questions and presenting them as clickable suggestions. Furthermore, it improves explainability by providing justifications, after code generation, for the selection of specific data representations and graph types most suitable for the query. Finally, as ChatMDV is built on MDV’s interactive design, it enables users to cross-query and filter data interactively across interlinked charts.

ChatMDV is a web-based platform that enables users to interactively visualise and further interrogate transcriptomics data using natural language within a unified analytical interface, and its framework can be extended to additional omics data types, including proteomics and genomics.

A comparison of existing tools highlights key differences in domain focus, deployment, visualisation capabilities, and interface design. OMEGA [19] for Napari [2] is primarily oriented toward imaging data and operates locally with a graphical interface that supports interactive visualisation. In contrast, CellAgent [12] and FlowAgent [11] focus on omics data and are also locally deployed but rely on Integrated Development Environments (IDEs) and CLI-based interfaces, respectively, with limited or no interactive visualisation capabilities. Notably, both CellAgent and FlowAgent incorporate LLM-driven automation for single-cell data analysis, enabling programmatic workflows rather than interactive exploration. ChatMDV similarly targets omics data but differs in combining local and web-based deployment with an interactive graphical interface and integrated visualisation capabilities. These distinctions emphasise that, while several tools support LLM-based automation, they vary substantially in how users engage with data, particularly in terms of interactivity and visual exploration. FlowAgent and CellAgent are designed to automate analytical workflows through sequential tool execution, whereas ChatMDV focuses on user-in-the-loop exploration through dynamic visual feedback. This distinction reflects complementary design goals rather than direct functional equivalence.

## Methods

### Design of ChatMDV

**Figure 1:**
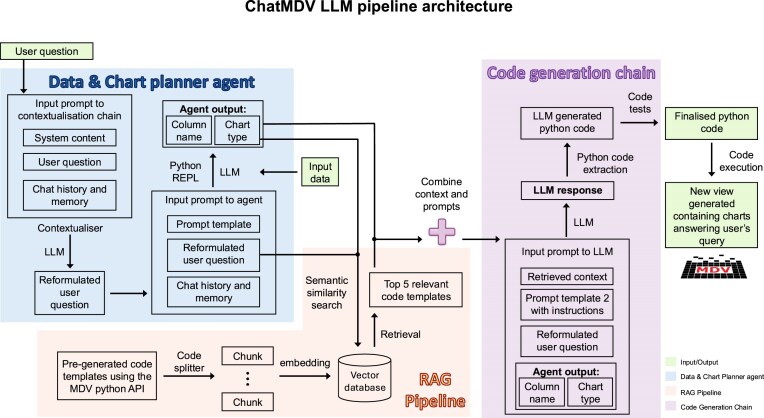
System Architecture of the ChatMDV Pipeline. ChatMDV comprises 3 core modules: the Data and Chart Planner Agent, the Retrieval Augmented Generation (RAG) Pipeline, and the Code Generation Chain. The Data and Chart Planner Agent module is responsible for interpreting user prompts, identifying the appropriate dataset components, and selecting the most suitable chart types for visualisation according to the dataset and question types. This agent also leverages conversational history to contextualise queries based on prior interactions, and it is able to autonomously call a Python Read-Eval-Print Loop (REPL) interface to execute code. The suggested chart types and the contextualised user prompt are then forwarded to the RAG Pipeline module. This module retrieves the 5 most relevant Python code templates based on similarity from a set of pregenerated code templates generated using the MDV Python Application Programming Interface (API) and stored in a vector database. Subsequently, the 5 most relevant templates, along with the identified dataset components, the suggested chart types, and the user’s contextualized query, serve as context input to the Code Generation Chain module. This module synthesises and executes the Python script generated. The resulting code and its corresponding view are then returned and rendered within the MDV interface, completing the user query with both executable code and visual output.

ChatMDV is composed of 3 modules that operate in sequence to interpret user queries and generate interactive charts visualised in MDV: the Data and Chart Planner Agent module, the RAG Pipeline module, and the Code Generation Chain module (Fig. 1). Collectively, these modules implement a combined agentic RAG framework optimised for adaptable, scalable, and reproducible biological data visualisation via structured task decomposition. Complex user queries are broken down into a sequence of manageable subtasks such as query interpretation, data field identification, chart type selection, code synthesis, and visualisation generation, which are handled by the specialised modules within the system. Additionally, memory and history functionality in the Data and Chart Planner Agent module support conversational context and continuity during multistep graph generation and iterative graph improvement (Fig. 1).

The LLMs supporting the framework require no fine-tuning. In this study, we evaluate multiple model backends (GPT-4o, GPT-4o-mini, GPT-4.1, and GPT-5-mini) to assess the impact of model capability on system performance.

MDV and ChatMDV can be deployed in a variety of ways depending on requirements. In general, we recommend installing within an isolated Docker container environment, which will allow interactive prompting and visualisation of the project using a standard desktop configuration (8–32 GB), even for large single-cell datasets (e.g., 1 million cells or more). If prompts trigger data analysis using Scanpy functions, memory requirements increase based on data size and type of Scanpy query used. In this case, deployment on high-memory systems with at least 128 GB RAM is recommended.

### Data and chart planner agent

This module initiates the pipeline by interpreting the user’s natural language prompt and reformulating it based on the conversation history and system context to maintain semantic continuity. For example, if a user first requests a uniform manifold approximation and projection (UMAP) scatterplot and then asks to colour it by gene expression, the follow-up query is contextually reformulated as follows: “Create a UMAP scatterplot coloured by gene expression.” Once the reformulated query is submitted, the custom LangChain [9] LLM-driven agent leverages its language comprehension capabilities and the Python Read-Eval-Print Loop (REPL) interface, an interactive environment for executing code in real time, to interrogate the dataset and identify the relevant data fields from the input. Additionally, instructed by the prompt framework, it recommends appropriate chart types based on the data fields’ data types. In summary, it performs 2 primary functions: (i) semantic parsing of the query and (ii) determination of the relevant data fields coupled with chart-type suggestions.

The prompt framework underpinning this module was based on the LangChain [9] documentation and the essential prompt elements manually crafted through iterative refinement and enhanced using ChatGPT [13]. These are available via the GitHub repository [20] for this project.

**Figure 2:**
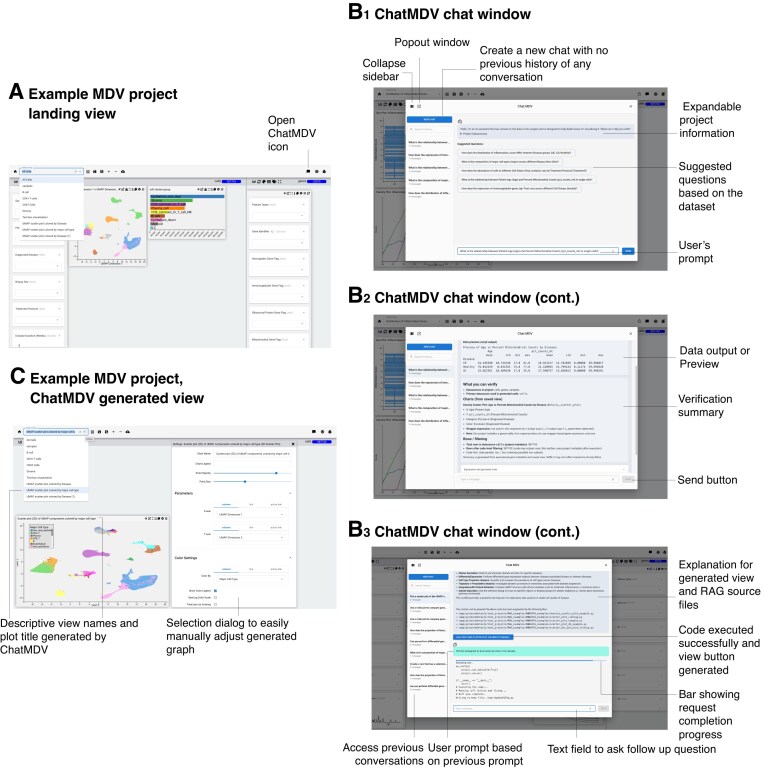
Multi-Dimensional Viewer (MDV) user interface (UI) and ChatMDV interaction flow. (A) Landing page of an MDV project displaying multiple charts, with a drop-down menu listing available views to browse. The ChatMDV icon is shown, and it is accessible for initiating an interactive session. (B_1_) Upon clicking the ChatMDV icon, a chat window appears with a collapsible sidebar and optional pop-out mode. The initial ChatMDV message appears, prompting users to ask a question. A set of dynamically generated example questions to ask ChatMDV is suggested, and information about the dataset, such as column names, is also shown. (B_2_) Users can submit natural language queries; conversation history is preserved, and a *new chat* button allows starting a fresh session. ChatMDV outputs a verification summary and information to help the user judge the veracity of the outputs. (B_3_) The Python code generated is accompanied by an LLM-generated explanation of plot choices and an outline of interpretive insights. Additionally, the file paths of the Python code templates used to inform ChatMDV’s context are given, providing interpretability. A progress bar indicates execution status of submitted queries, along with real-time feedback on ChatMDV’s reasoning. The *view* button becomes visible after scrolling. Previous conversations can be accessed through the sidebar. (C) The generated visualisations appear upon clicking the *new view* button. Descriptive view names and plot titles aid interpretation, adding an extra layer of information helping the user comprehend the content of the view. The MDV point-and-click interface further supports interactive chart editing, enabling even more flexibility for the user.

An example prompt (seen in Fig. 2) is “Plot a scatterplot of the UMAP components.” This guides the module to search the dataset for the “UMAP components.” As the agent has access to the dataset, it can search it in the Python REPL environment using the Pandas package [21] and retrieve the exact column names corresponding to the UMAP components. Because the prompt specifies a particular chart type (scatterplot), the agent also returns “scatterplot” as a visualisation suggestion, alongside the relevant column names. This allows the agent to provide the exact column names and minimises hallucinations. If the initial column retrieval is not satisfactory to the agent, the agent retries using an iterative refinement strategy until it returns a satisfactory output or reaches the maximum iteration limit.

By generating a targeted list of columns grouped by visualisation intent, this module significantly reduces overhead and irrelevant data handling downstream.

### Retrieval augmented generation pipeline

To populate the vector database in the RAG pipeline module, we developed a library of Python code templates, 1 per chart type supported by MDV, along with additional templates that combine multiple different chart types. The pregenerated Python code templates use the MDV Python Application Programming Interface (API), an API that enables technically proficient users to programmatically construct, customize, and deploy their MDV projects composed of interactive views created based on their datasets. These act as data-agnostic templates, ensuring compatibility across diverse datasets and promoting generalisation, reusability, and flexibility. Each code template is segmented into modular code chunks and embedded into vector representations (Fig. 1). These embeddings are indexed within an open-source FAISS vector database [22], enabling fast retrieval of relevant code snippets at inference time (Fig. 1). The 5 templates most semantically similar to the reformulated user question and the suggested chart types are retrieved and passed as contextual information to the Code Generation Chain module (Fig. 1). This architecture focuses the LLM responses to MDV API compatible code, minimises hallucinations, and provides a flexible mechanism to support new chart types as the MDV architecture evolves eliminating the need for fine-tuning the LLM. Continuing with the example prompt, “Plot a scatterplot of the UMAP components,” this query and the structured output from the previous module are passed to the RAG pipeline module. In response, the module retrieves the top 5 most semantically relevant Python code templates from the indexed repository, which in this case includes templates associated with scatterplot visualisations.

The RAG retrieval component is essential for grounding the model in the MDV Python API, which is not part of the standard training distribution of general-purpose LLMs. Without retrieval of API-aligned code templates, the model is unlikely to generate syntactically valid or executable MDV-compatible code.

### Code generation chain

Following the identification of relevant data fields, the chart types suggestion, and the selection of the most semantically similar pregenerated Python code templates, this information is passed as contextual input to the subsequent Code Generation Chain module. This module employs a context-augmented generation strategy to synthesize executable Python visualisation scripts, based on the MDV API. By incorporating the retrieved data fields, recommended chart types, and representative MDV API-based code templates into the prompt, the system is guided to produce code that is both semantically aligned with the user’s intent and syntactically compatible with the MDV framework (Fig. 1). For example, for the query “Plot a scatterplot of the UMAP components,” the scatterplot template passed as context will be prioritised by the LLM and the generated code modelled on the template. The UMAP-related column names will be used along with customised view names, title names, and chart colours.

Using the augmented prompt, the LLM generates a response containing a Python code script based on the pregenerated templates. The resulting script contains dataset-specific fields, adjusted axis labels and chart titles, a comprehensive view name, and dataset path and addresses the visualisation request put forward by the user. The next step in the module sequence is Python code extraction, as seen in Fig. 1, which is performed using a bespoke script run on the server. The script is executed on an isolated Python process within the MDV hosting environment, and standard output (successful run) and standard errors are captured. The resulting execution logs are also obtained. The final Python script generates output in JSON format, compatible with MDV rendering.

The system employs a custom Python execution harness that serializes the target code to a temporary file, executes it in an isolated Python subprocess, and captures standard output and error streams. The resulting execution logs are then programmatically evaluated to verify successful completion within the MDV hosting environment.

This enables visualisations to be served interactively within the MDV interface, supporting direct manipulation of charts and sharing. The Code Generation Chain module primarily synthesizes Python scripts aligned with the MDV API, guided by context augmentation from relevant data fields, chart types, and pregenerated Python code templates. While the code generation uses the MDV framework, the underlying LLM also has access to widely used Python-based single-cell gene expression analysis libraries such as Scanpy [18]. As a result, when user queries require additional analytical steps, such as identifying highly differentially expressed genes, the model can incorporate these operations into the generated script while maintaining compatibility with the MDV framework. The choice of Python as the target language further enables integration with a broad ecosystem of scientific libraries, enhancing the analytical flexibility and extensibility of the platform.

Together, these modules allow ChatMDV to convert biological questions into visual answers: automatically, interactively, and reproducibly.

### MDV user interface and ChatMDV

ChatMDV is fully integrated into the MDV user interface, enabling users to combine the flexibility of natural language–driven view generation with MDV’s interactive visualisation environment. This integration allows users not only to generate initial visualisations using the interactive chat interface but also to further refine or extend them using MDV’s native tools, creating additional views or modifying existing ones for deeper data exploration. This combination of executable code, dataset summaries, and interactive visualisation provides a transparent workflow that allows users to verify that generated outputs align with their intended query. To support result verification, ChatMDV provides a “Verification summary” for each generated view. This summary reports the data sources, chart types, parameters used, and any filtering operations, allowing users to confirm that the generated visualisation aligns with their intended query without inspecting the full underlying code (see Fig. 2B).

To use ChatMDV, a user clicks on the ChatMDV icon on the top right of the user interface when in a project (Fig. 2A). ChatMDV automatically generates a set of suggested questions, with the help of LLMs, to provide a starting point for interaction and provide ideas for dataset exploration (Fig. 2B_1_). To further help the user understand the dataset, we show an expandable summary of the data columns, types, and value ranges (Fig. 2B_1_). Once the user asks a question, they can submit the request using the send button (Fig. 2B_2_). ChatMDV shows a progress bar along with other useful information regarding the advancement of the query (Fig. 2B_3_). Following successful output generation, the Python code is displayed and a view button is generated, which directs the user to the new view containing the requested chart(s). As part of the output, an explanatory text is provided describing why the generated charts were used, how they can be interpreted, and future questions to address the user’s query. In addition, the file paths of the pregenerated code templates retrieved are returned, allowing users to trace which scripts were incorporated into the LLM’s context during generation. In addition to chart-based outputs, ChatMDV also renders direct textual analytical responses in the chat window. This helps the user maintain flow and is useful for prompts that express analytical intent, such as returning results from scanpy functions.

The ChatMDV pipeline architecture is designed to maintain awareness of conversation context, enabling a user to ask follow-up questions in a conversational manner incorporating conversation history. For example, after generating a view, a user can ask subsequent queries to modify the visualisations, such as asking to add additional genes in a gene expression plot without needing to restate the full request (Fig. 2B_3_). Previous conversations can be accessed through a collapsible sidebar on the left-hand side of the user interface (UI) (Fig. 2B_3_). MDV’s rich graphical interface allows users to customize output visualisations through various chart settings, including point size, colour scheme, axis dimensions and font size, and legend position (Fig. 2C).

### Instance and project creation

MDV instances can be locally installed by following the instructions in the GitHub repository [20]. ChatMDV can be enabled by providing an OpenAI API key. Guidance and instructions can be found on our website [23] and in our GitHub repository [20]. An example dataset is provided on our website, along with more detailed instructions on uploading your own dataset.

Once an MDV instance is set up, the scRNA-seq dataset or project can be uploaded to MDV. An scRNA-seq dataset is usually stored as an Anndata object [17], a data structure used in single-cell analysis that stores the gene expression matrix along with cell- and gene-level metadata. Linking the gene expression matrix with the associated cell and gene metadata is performed automatically as part of the MDV project creation, and a user can access both the cells’ and genes’ dimensions stored in the object in MDV. Therefore, a user can access the data and generate more complex, biologically relevant, and informative charts. Gene identifiers such as gene IDs or gene names are used to link the data.

## Evaluation and Case Study

### Datasets for ChatMDV evaluation

To demonstrate the usability and practical utility of ChatMDV in data exploration and visualisation, we selected scRNA-seq as a representative modality. scRNA-seq datasets are characterized by their high dimensionality, cellular diversity, and increasing ubiquity in modern biomedical studies, making them an ideal benchmark for assessing the capabilities of ChatMDV. To this end, we selected three publicly available scRNA-seq datasets of varying biological and technical complexity, which served as the basis for constructing domain-relevant queries to evaluate ChatMDV’s functionality.

The first dataset is the widely used Peripheral Blood Mononuclear Cells 3K dataset (PBMC3K) [24–26]. It includes transcriptomic profiles from 2,700 immune cells isolated from a healthy donor. This benchmark dataset has a manageable size and well-characterised annotations, making it an ideal test bed for validating ChatMDV’s core visualisation capabilities, including scatterplots of UMAP projections, gene expression heatmaps, and violin plots. The second dataset is a comprehensive lung cancer atlas from the Human Cell Atlas [27, 28], offering a rich landscape of cell states across healthy and diseased lung tissue at a single time point. It includes 2,282,447 cells from the respiratory tract of 486 individuals [27, 28]. The third dataset is the TAURUS dataset [29, 30], which comprises longitudinal scRNA-seq profiles from patients with Crohn’s disease (CD) and ulcerative colitis (UC) undergoing anti–tumour necrosis factor (anti-TNF) therapy. It captures samples from pre- and posttreatment time points and includes subclustered cell states annotated across multiple disease and response groups for 987,743 cells. Each dataset introduces an additional layer of complexity. The PBMC3K [24–26] dataset is a relatively small and well-characterised reference dataset. The lung cancer atlas dataset [27, 28] adds complexity through its cross-sectional design, capturing cells from multiple individuals and tissue/cell types. The TAURUS [29, 30] dataset adds a longitudinal component to the cross-sectional design by collecting samples from multiple individuals at multiple time points, both before and after anti-TNF treatment. This enables the construction of more advanced, biologically contextualised queries to prompt ChatMDV for each dataset.

### Evaluation approach

**Figure 3:**
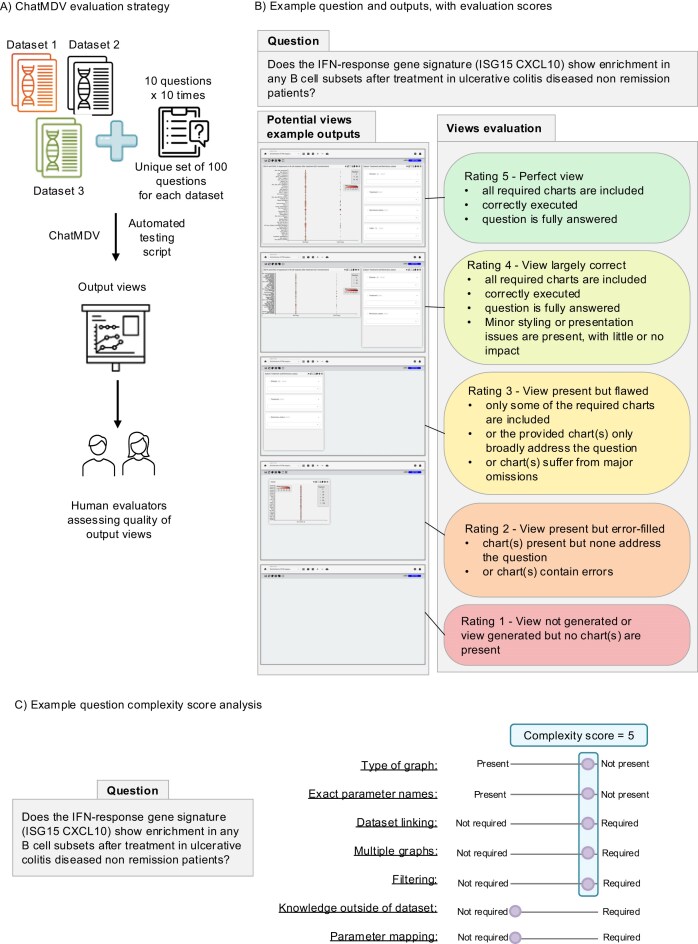
ChatMDV evaluation strategy, evaluation example, and complexity score assignment example. (A) ChatMDV was evaluated using 3 distinct scRNA-seq datasets to demonstrate its generalisability and broad applicability. For each dataset, a set of 10 unique natural language questions was curated, and each question was submitted 10 times using an automated Python evaluation testing script. The resulting visualisations were generated within the same MDV project, assessed, and scored. (B) Example of a question submitted to ChatMDV, with representative visualisation outputs and corresponding qualitative evaluation ratings. (C) Complexity analysis and complexity score assignment example for the example question presented in B.

To validate ChatMDV’s capabilities, we developed a systematic approach for testing and assessing the system’s performance. We developed a set of domain-specific questions to simulate typical exploratory biological analysis with the goal of elucidating data properties and aiding in scientific discovery and hypothesis generation. Natural language prompts were written to resemble domain-specific questions a typical biologist might pose, such as comparing gene expression across cell states or visualizing treatment effects over time or for different patients. As part of the assessment, an automated evaluation system was built to submit the set of user queries to ChatMDV and generate responses addressing each query as distinct views within a new MDV project for each dataset (Fig. 3A). The generated views were then manually reviewed and annotated and assigned ratings on a 5-point scale, ranging from 1 (view not generated or no charts present) to 5 (perfect view). These ratings are summarised in Fig. 3B and described in detail in “Output classification and evaluation criteria.” Additionally, each question was assigned a complexity score (ranging from 1 to 7), reflecting its semantic and analytical difficulty, which was found to impact ChatMDV’s performance. Further details on the complexity scoring scheme are provided in “Prompt design and complexity categorisation” and also seen in Fig. 3C. This primary benchmark was intentionally constructed using prompts to ensure biological relevance, coverage of supported chart types, and controlled variation in complexity. We note that such prompts may be better formed than those produced by novice or occasional users, who may provide underspecified requests, ambiguous phrasing, or analysis-oriented questions without explicit visualisation instructions.

Because ChatMDV is implemented as an integrated multistage pipeline, where intermediate components (e.g., intent interpretation, retrieval, and code generation) are interdependent, we prioritise end-to-end evaluation. In this setting, correctness is defined by the final generated visualisation rather than intermediate outputs in isolation. However, to provide additional insight into system behaviour, we include stage-aware diagnostic analyses based on error types and complexity factors (see Supplementary Fig. S1 and Supplementary Data Table for Fig. S1).

Each complexity criterion also corresponds to different stages of the ChatMDV pipeline. For example, parameter identification and mapping primarily affect the Data and Chart Planner Agent, while execution success reflects the behaviour of the Code Generation Chain. This mapping allows indirect assessment of stage-specific challenges without isolating modules from the end-to-end workflow.

### Prompt design and complexity categorisation

**Figure 4:**
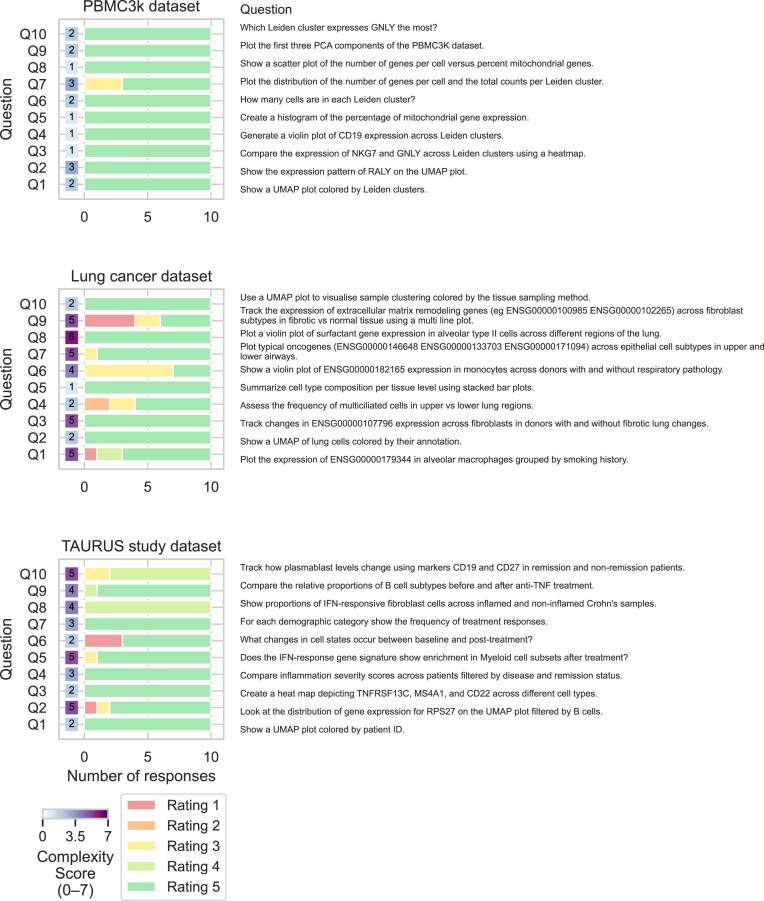
ChatMDV evaluation results. Summary of evaluation outcomes, shown for each dataset. Evaluation questions are shown on the right-hand side of the y-axis with the corresponding complexity score on the left-hand side. Each question is run through the automated evaluation script 10 times, and the outcomes are annotated using a rating scale, ranging from “rating 5” (which corresponds to a “perfect view”) to “rating 1” (corresponding to an empty view or a view that is not generated). Next to the evaluation outcomes barplots, the complexity score is shown, with the most complex question being assigned a value of 7 and the least complex being assigned a value of 1. Detailed complexity scoring criteria for each question and complexity assignment are provided in Fig. 3 and Supplementary File 1. The breakdown of the annotation results per dataset is provided in Supplementary File 3.

Multiple test prompts were developed for each dataset to ensure coverage of a broad range of biological use cases. The prompts (which are reported in Fig. 4 and in Supplementary File 1) were developed through a combination of bioinformatic expert consultations, a detailed review of the relevant scientific literature accompanying each dataset, and ChatGPT’s assistance [13]. During the creation process, we ensured that the prompts were biologically relevant, spanned a range of charts supported by MDV, and varied in complexity. We assessed the complexity of each test prompt by using a bespoke complexity schema. We assigned a complexity score to each test prompt by assigning a point to the overall score based on whether the prompt meets the following criteria:

1. **Type of graph:** The prompt explicitly states the desired graph type (e.g., bar chart, line plot, scatterplot). Omitting this information increases the complexity score by 1 point.
2. **Exact parameter names:** The prompt includes the precise column or gene names as they appear in the dataset, including case sensitivity, for all the required data fields. If exact field names are not provided, a complexity point is added.
3. **Dataset linking:** If the prompt requires combining information from different data sources (e.g., linking gene expression data with metadata using gene IDs), 1 point is added.
4. **Multiple graphs:** The prompt expects the generation of more than 1 graph, which adds an additional complexity point.
5. **Data filtering:** If the prompt includes conditions that require filtering or selecting specific subsets of the data (e.g., only patients with a certain disease), 1 point is added.
6. **Knowledge outside of the dataset:** If the prompt refers to information not directly available in the dataset (e.g., referencing a class of genes without listing them), it adds complexity.
7. **Parameter mapping:** The LLM has to identify that the information in the prompt requires mapping to a different representation from the dataset. This additional reasoning step adds to the prompt’s complexity. For example, if a gene is referenced by name in the prompt but is stored as an Ensembl ID in the dataset [31], we assign an additional complexity point.

The complexity score is a descriptive additive index rather than a weighted model. Each criterion contributes equally to maintain interpretability and avoid overfitting, given the evaluation set size.

Ten questions were constructed for each dataset, and each one was submitted to ChatMDV 10 times, resulting in a set of 100 natural language prompts and outputs to be evaluated (Fig. 3). For each question, ChatMDV’s output was stored. This included the “Data and Chart Planner Agent” module’s output, the top 5 relevant code templates retrieved and passed as context, and the output code generated and executed by ChatMDV. Potential execution errors were also recorded for further understanding, debugging, and improving the pipeline. The full output of ChatMDV for each question is given in Supplementary File 2.

This evaluation pipeline allowed us to systematically assess ChatMDV’s capacity to understand varying query structures and intent. It helped us assess the reproducibility of the results and the robustness of the system. Moreover, it helped improve the pipeline by prompt engineering and experimenting with both the agent’s and RAG systems’ parameters. The iterative improvement process was performed with different prompts, which spanned from very low-complexity prompts such as “Plot a scatterplot of the number of genes versus the number of cells” and reached high-complexity prompts such as “Track how plasmablast levels using expression markers such as CD19 and CD27 change from before to after treatment in Crohn’s disease patients for patients in remission and nonremission.”

### Output classification and evaluation criteria

The output of the evaluation pipeline was manually evaluated using a rating system to assess the completeness, quality, and relevance of each generated view (Fig. 3). A rating of 5, “perfect view,” was assigned to the view when all required charts were present, and the code generating them correctly executed and fully answered the natural language prompt. A rating of 4, “view largely correct,” indicated that all necessary charts and data were included and accurate, but with minor clarity issues or styling problems. For example, a font might be difficult to read on a chart axis—something that can easily be remedied as a user by manipulating the chart properties in the MDV user interface. A rating of 3, “view present but flawed,” reflected partial success; for example, only some required charts were present, or the charts only broadly addressed the question or suffered from major omissions. A rating of 2, “view present but error-filled,” was given when charts were present but did not address the question or contained significant errors. Finally, a rating of 1 indicated that no view was generated or an empty view was generated without any visual content. Common reasons for this included failure to generate Python code (i.e., the pipeline did not complete), Python code generation followed by execution errors, or successful execution of Python code that failed to produce valid graphs due to runtime errors. Summarising the results, error classification and investigating the reasons behind failed chart/view generation enabled targeted system improvements and informed development priorities. These were addressed using prompt engineering, optimising conditions related to the RAG pipeline such as the similarity threshold, embeddings model choice, model temperature, and improved error handling and recovery mechanisms. The underlying reasons for not achieving a rating of 4 or 5 included incorrect identification of variable names corresponding to the desired parameters, mismatches between the data type required by the chart type used, failure to recognise and map relevant genes present in the dataset, and omission of selection dialog plots necessary for filtering the data.

## Results

A user can instruct ChatMDV to “plot a chart of the UMAP components” of a dataset, and ChatMDV can choose a suitable graph (scatterplot) and visualise the user’s request (Supplementary Video 1). Moreover, ChatMDV is able to identify the column names from within the dataset that correspond to the “UMAP components” so it can create a visualisation successfully (Supplementary Video 1). Users can ask follow-up questions to modify the visualisation (Supplementary Video 2), utilising ChatMDV’s capability of retaining conversational history. ChatMDV can also create more than 1 visualisation within the same view by asking it, “Can you plot the 20 most important genes in the dataset and show their expression in box plots against the lung tissue?” (Supplementary Video 3). This functionality is particularly useful for automating repetitive graph generation tasks such as creating the same plot type for multiple marker genes. The generated charts can then be interactively adjusted and customised by the user within the MDV interface (Supplementary Video 3). The datasets within the project can be filtered and subsetted, for example, by asking ChatMDV to “Compare inflammation severity scores across patients filtered by disease and remission status.” ChatMDV adds a selection dialog plot, which is MDV’s chart type for filtering the dataset (Supplementary Video 4). Adding a text box is a chart functionality supported by MDV. ChatMDV is able to utilise this type of chart to output information about the dataset (Supplementary Videos 5 and 6) and beyond. Example natural language prompts include “Print your understanding about the dataset in a text box” and “Print summary statistics and other important info about the dataset in a text box” (Supplementary Videos 5 and 6, respectively). scRNA-seq data are stored in Anndata objects [17], which organise information into multiple matrices, usually separating cell metadata and gene annotations into distinct structured components. Plotting charts showing gene expression-related properties requires integrating the cells’ and genes’ matrices by linking on gene identifiers. ChatMDV’s capabilities involve linking different matrices on predefined gene identifiers (Supplementary Video 7). The linking gene identifier is predefined in the Anndata object, and it can be one of gene name or gene ID. ChatMDV can accurately map between gene names and gene IDs, ensuring that the appropriate data are retrieved and the correct plots are generated (Supplementary Video 8). The lung cancer dataset uses Ensembl gene IDs [31] as the gene identifier, and in Supplementary Video 8, the natural language prompt (“Plot the expression of HLA-DQB1 in alveolar macrophages grouped by smoking history”) is querying ChatMDV using the gene name.

By implementing the ChatMDV pipeline with support for executing Python code, we enable the underlying LLM to apply its external domain knowledge and make use of established packages for scRNA-seq analysis such as Scanpy [18], a widely used package in the field of single-cell analysis. In Supplementary Video 9, ChatMDV demonstrates this capability by invoking the rank_genes_groups() function to perform differential gene expression analysis, filter statistically significant genes, and identify top candidates based on effect size. It then visualises the expression patterns of the top 3 genes across cell clusters using both a dot plot and a heatmap plot. The example demonstrated in Supplementary Video 9 uses the PBMC3K dataset, whose .h5ad file is approximately 32 MB in size. In comparison, the TAURUS and lung cancer datasets are 3 orders of magnitude larger. As discussed, running Scanpy functions on such large datasets often requires significant amounts of random-access memory (RAM) due to the way scanpy works rather than ChatMDV.

### Evaluation of ChatMDV performance

To evaluate ChatMDV’s performance and robustness, we used the evaluation pipeline described in “Prompt design and complexity categorisation” and “Output classification and evaluation criteria.” A summary of results is presented in Fig. 4, with a detailed breakdown available in Supplementary File 3. For each dataset, a set of 100 prompts was used as an independent test set and was not part of the iterative refinement process.

To provide a clearer assessment of performance, we distinguish between three evaluation measures: execution success (ratings 2–5), semantic success (ratings 4–5), and perfect success (rating 5), where all required charts are present and the query is fully satisfied. A summary of these metrics across datasets is provided in Table 1.

**Table 1:** Summary of ChatMDV performance across datasets. Execution success corresponds to ratings 2–5, semantic success to ratings 4–5, and perfect success to rating 5.

Dataset	Execution success (2–5), %	Semantic success (4–5), %	Perfect success (5), %	Mean rating
PBMC3K	100.0	97.0	96.0	4.90
Lung	96.0	79.0	79.0	4.39
TAURUS	97.0	92.0	73.3	4.52

We rank the datasets by increasing biological and structural complexity as PBMC3K, lung, and TAURUS. Performance decreases with increasing dataset complexity, reflecting factors such as dataset size, number of available columns, and variability in annotation quality.

We observe that all natural language prompts receiving a rating of 1 had a complexity score greater than or equal to 2. This indicates that failures are associated with prompts that require additional interpretation, such as missing parameter names, filtering, or mapping steps. In contrast, simpler prompts that explicitly specify chart types and parameter names consistently achieve semantic success (ratings 4–5).

Performance varies across datasets (see Table 1), with PBMC3K showing near-perfect outcomes and reduced performance observed for the lung cancer and TAURUS datasets. This trend is consistent with increasing dataset complexity, including differences in dataset size (PBMC3K <100 MB; lung cancer $\sim$22 GB; TAURUS $\sim$12 GB) and the number of available columns (PBMC3K: 12, lung cancer: 76, TAURUS: 42), which increase the search space for the Data and Chart Planner Agent.

To further characterise these effects, we analysed the impact of individual complexity factors by reporting a complexity-weighted mean manual rating, computed by weighting each prompt by its complexity score. This provides a summary that accounts for variation in task difficulty without introducing fitted weighting schemes (see Supplementary Fig. S1 and Supplementary Data Table for Fig. S1). This shows that ambiguity-related factors (such as missing graph type, filtering requirements, and parameter mapping) have the largest negative impact on performance, particularly in the lung cancer dataset. In contrast, simpler datasets such as PBMC3K are more robust to these effects.

We also note that dataset-specific characteristics, such as the consistency and clarity of column naming, influence performance. For example, the TAURUS dataset includes more structured and descriptive annotations, whereas the lung cancer dataset contains less consistently curated column names, which may contribute to increased ambiguity and error rates.

### Qualitative evaluation of novice-style prompts

To explore how ChatMDV behaves on less structured real-world inputs, we conducted a small-scale qualitative evaluation using prompts provided by 3 novice users. Compared with the curated benchmark, these prompts were more often underspecified, omitted chart-type instructions, or expressed analysis goals rather than direct visualisation requests (e.g., “Give me the top 5 marker genes in each cluster,” “Can you summarize how many cells in each Leiden cluster?” “Can you get me top 10 differentially expressed genes across all the clusters?”).

This evaluation showed that novice prompts frequently require the system to infer whether the intended output should be a visualisation or a textual analytical summary to align with the user’s intent. In earlier versions, ChatMDV tended to generate visual outputs even when a textual response would have been more appropriate, which could result in empty or uninformative views unless they specified to output results in a text box. We therefore updated the system to support direct rendering of textual responses within the ChatMDV interface when the user query is better matched to a nonvisual analytical output. This improves robustness to novice-style prompts and better aligns system behaviour with user intent.

### Evaluation of alternative model performance

To assess the impact of model capability, we evaluated ChatMDV using multiple LLM backends (GPT-4o, GPT-4o-mini, GPT-4.1, and GPT-5-mini). For each model, we report execution success (ratings 2–5), semantic success (ratings 4–5), and perfect success (rating 5) across all datasets (see Supplementary Fig. S2 and Supplementary Data Table for Fig. S2).

We observe that lower-capability models (e.g., GPT-4o-mini) exhibit substantially reduced semantic and perfect success rates, while higher-capability models (e.g., GPT-4.1 and GPT-5-mini) achieve consistently higher performance but with increased latency. These results indicate that model capability plays a critical role in enabling accurate and reliable visualisation generation within the ChatMDV pipeline.

## Discussion

ChatMDV introduces a novel approach to biological data visualisation using natural language. Building on MDV’s established visualisation functionality, it provides a compelling way to explore large and complex single-cell datasets. The increasing application of AI across genomics further highlights the need for accessible interfaces to complex analytical workflows [32]. Using LLMs and a sequential RAG pipeline, ChatMDV suggests data-driven questions to serve as a starting point for analysis. Biological questions such as “Which Leiden cluster expresses GNLY the most?” and “Run a differential expression analysis to find key genes” can be used, allowing users to focus on intent rather than learning complex user interfaces or coding. The design reflects the Findability, Accessibility, Interoperability, and Reuse (FAIR) principles [33] of data management:

**Findability:** ChatMDV integrates seamlessly with MDV’s structured project and metadata framework, ensuring that visualisations, code, and datasets are systematically catalogued and retrievable. Each generated view is linked to its executable Python code, maintaining transparent provenance and clear traceability. Our RAG approach also enhances query transparency by linking generated content back to the source code. This traceability improves user confidence and interpretability, which is an important factor for adoption in scientific workflows.

**Accessibility:** The system’s natural language interface reduces the barriers to access to advanced visualisation and analysis tools. Users without programming expertise can perform complex analyses through intuitive dialogue, while the Docker deployment ensures consistent, platform-independent accessibility. The inclusion of explicit verification summaries further reduces the cognitive overhead of validating results, helping ensure that the cost of checking LLM-generated outputs remains lower than manual workflow alternatives.

**Interoperability:** ChatMDV leverages standard Python data structures (e.g., AnnData) and interoperates with widely adopted bioinformatics packages such as Scanpy [18] and future integrations of other libraries such as Squidpy [34]. The modular RAG framework facilitates integration with other LLM-enabled agents and analytical ecosystems, enabling smooth data exchange and workflow extension.

**Reusability:** Every query generates reproducible code that can be inspected, shared, or re-executed. This explicit linkage between natural language commands, code, and output ensures scientific transparency and facilitates reuse across datasets, users, and research contexts.

MDV’s user interface allows postrefinement of the chart properties (layout, font size, etc.). This provides a seamless workflow to query data and produce visualisations that can be used in presentations and publications. By coupling LLM-driven automation with point-and-click refinement, ChatMDV makes complex biological data exploration both accessible and customisable for users with varying levels of computational expertise.

Collectively, these features make ChatMDV not only a powerful analytical companion but also a platform aligned with the FAIR principles [33], ensuring that biological data visualisation is accessible, transparent, and sustainable for the broader research community. By uniting automation, interpretability, and openness, ChatMDV exemplifies how LLM-based systems can support reproducible and equitable science.

### Future directions

Tools like FlowAgent [11] show how multiagent planners can automate scRNA-seq pipelines and general bioinformatics workflows. ChatMDV slots into this ecosystem as the interactive visual layer, which in future could enable a seamless transition from agent-generated results to user-driven visual exploration. In this fast-moving field, we predict LLM services around biological data types will rapidly increase. Frameworks such as the Model Context Protocol (MCP) [35] offer a public, language–agnostic specification that any agent can plug into, improving discoverability and cross–tool portability. Services such as SCMCP hub [36] and MCPmed [37] can be incorporated into MDV’s framework to allow a range of diverse services to be visualised based on natural language.

We see ChatMDV’s RAG architecture facilitating ways of integrating other unstructured biological assets, including PDFs, Electronic Lab Notebooks, and Jupyter notebook snippets, allowing the system to suggest domain-aware queries building on the automated suggested queries already available.

Finally, open-source LLMs, such as LLaMA [7], can be used for local deployment, rather than using commercial AI APIs, prioritising data privacy for groups or organisations that wish to use ChatMDV to interact with sensitive patient data.

### Limitations

Occasionally, the system may select data types that are not optimal for a given chart. For example, assigning a categorical variable to a parameter that expects numerical input, such as in scatterplots or line plots. Other examples include when ChatMDV hallucinates a parameter name or tries to use a gene that does not exist in the dataset. To address this, we implemented extensive error handling, iterative retries, and comprehensive logging to help identify and resolve issues quickly.

Other failures can occur when there is a mismatch between the user’s understanding of the dataset and how the project is indexed or annotated. For example, a user may request a gene by symbol when the dataset is indexed by Ensembl gene ID, which can lead to empty or incorrect outputs if the mapping is not resolved. Similarly, ambiguous or poorly curated column names can cause incorrect field selection or failure to generate the intended visualisation. These issues are primarily driven by dataset metadata quality and could be reduced through clearer field naming, better annotation, and explicit identifier mappings.

When ChatMDV is required to perform more memory-intensive scRNA-seq analyses (e.g., using Scanpy), failures may occur due to the need to load large data matrices into RAM. These limitations arise from the underlying analysis libraries rather than the MDV visualisation layer or ChatMDV interface and can be mitigated by running on more powerful hardware.

## Availability of Source Code and Requirements

- **Project name:** ChatMDV
- **Project page:**  https://github.com/Taylor-CCB-Group/MDV
- **Operating system(s):** Platform independent (tested on Linux and macOS)
- **Programming language:** Python/Javascript/Typescript
- **Other requirements:** OpenAI API key, MDV (latest release), Docker is recommended (for local install)
- **License:** GPL-3.0 License
- **Containerisation:** A Docker container supporting the full pipeline is available in our GitHub repository, https://github.com/Taylor-CCB-Group/MDV
- **Restrictions to use by nonacademics:** None

## Additional Files

**Supplementary File 1**. Contains the full set of natural language prompts used to evaluate ChatMDV across all 3 datasets, along with the corresponding complexity scores. Additionally, contains a table indicating which complexity criteria are fulfilled for each prompt, with crosses denoting satisfied conditions.

**Supplementary File 2**. Contains the ChatMDV output per prompt for each dataset shown in Fig. 4. The file includes the output of the “Data and Chart Planner Agent,” the top 5 relevant code templates retrieved and passed as contexts, and the output code generated by ChatMDV for each natural language prompt. In cases where a view failed to be generated, corresponding code execution errors are also reported.

**Supplementary File 3**. Contains the detailed breakdown of the evaluation results for each dataset summarised in Fig. 4. Annotated rating is given for each prompt run through the evaluation system.

**Supplementary File 4**. Contains the data used to generate the figure in Supplementary Fig. S1 assessing the effect of complexity factors on ChatMDV performance across the test datasets.

**Supplementary Figure S1**. The effect of complexity factors on ChatMDV performance across the test datasets.

**Supplementary Figure S2**. Performance of ChatMDV across different LLM models.

**Supplementary Video 1**. By interpreting the user’s intent, ChatMDV selects appropriate chart types and parameter names to generate a suitable graph. The video has been sped up by a factor of 2 and includes a voiceover and subtitles.

**Supplementary Video 2**. ChatMDV allows users to interactively modify the generated visualisations via follow-up questions referencing the previously generated graph. The video has been sped up by a factor of 2 and includes a voiceover and subtitles.

**Supplementary Video 3**. A single natural language query can prompt ChatMDV to produce several charts within the same view. Once generated, the user can further refine and modify the individual charts interactively within the MDV interface. The video has been sped up by a factor of 2 and includes a voiceover and subtitles.

**Supplementary Video 4**. ChatMDV enables interactive dataset filtering by generating selection dialog plots in response to natural language prompts. The video has been sped up by a factor of 2 and includes a voiceover and subtitles.

**Supplementary Video 5**. ChatMDV uses MDV’s text box chart functionality to produce textual summaries describing the dataset. The video has been sped up by a factor of 2 and includes a voiceover and subtitles.

**Supplementary Video 6**. ChatMDV generates summaries of dataset statistics using MDV’s text box chart type. The video has been sped up by a factor of 2 and includes a voiceover and subtitles.

**Supplementary Video 7**. ChatMDV is able to link data from distinct matrices stored in the Anndata object. In this example, the linking of gene expression is demonstrated by colouring the scatterplot showing the UMAP components by the expression of the *RPS27* gene for the TAURUS dataset. The video has been sped up by a factor of 2 and includes a voiceover and subtitles.

**Supplementary Video 8**. Not all linking identifiers are gene names; they can also be gene IDs. In this example, ChatMDV automatically determines the correct linking identifier, gene ID in this case, even though the user’s prompt refers to the gene by name, demonstrating its ability to map between different linking fields. The video has been sped up by a factor of 2 and includes a voiceover and subtitles.

**Supplementary Video 9**. ChatMDV performs differential gene expression analysis using rank_genes_groups() from Scanpy [18], filters significant genes by effect size, and visualises the top 3 expressing genes across cell clusters using a dot plot and heatmap. The video has been sped up by a factor of 2 and includes a voiceover and subtitles.

**Supplementary Data Table for Figure S1**. Data used to generate Supplementary Figure S1.

**Supplementary Data Table for Figure S2**. Data used to generate Supplementary Figure S2.

## Abbreviations

AI: artificial intelligence; anti-TNF: anti–tumour necrosis factor; APIs: application programming interfaces; CD: Crohn’s disease; CLIs: command line interfaces; FAIR: Findability, Accessibility, Interoperability, and Reuse; GUIs: graphical user interfaces; IDEs: Integrated Development Environments; LLMs: large language models; MCP: Model Context Protocol; MDV: Multi-Dimensional Viewer; NLPs: natural language processing; PBMC3K: Peripheral Blood Mononuclear Cells 3K; RAG: retrieval augmented generation; RAM: random-access memory; REPL: Read-Eval-Print Loop; scRNA-seq: single-cell RNA sequencing; UC: ulcerative colitis; UI: user interface; UMAP: Uniform Manifold Approximation and Projection.

## Supplementary Material

giag073_Supplemental_Files

giag073_Authors_Response_To_Reviewer_Comments_original_submission

giag073_GIGA-D-25-00446_original_submission

giag073_GIGA-D-25-00446_revision_1

giag073_Reviewer_1_Report_original_submission Reviewer 1 -- 12/17/2025

giag073_Reviewer_1_Report_revision_1 Reviewer 1 -- 5/11/2026

giag073_Reviewer_2_Report_original_submission Reviewer 2 -- 1/14/2026

giag073_Reviewer_2_Report_revision_1 Reviewer 2 -- 5/2/2026

## Data Availability

All datasets used in this study are publicly available and have been previously published. The PBMC3K dataset is available from 10x Genomics [24–26], the lung cancer atlas dataset from the Human Cell Atlas [27, 28], and the TAURUS dataset [29, 30] from the original study authors. Accession numbers and links to these datasets are provided in the reference list. No new datasets were generated in this study.
