# Supplementary material for: ChatMDV: reducing technical barriers in bioinformatics analysis using large language models
Source: Gigascience. 2026 Jun 19;15:giag073. doi: 10.1093/gigascience/giag073 (PMC13452166; doi:10.1093/gigascience/giag073)
Supplement: Supplementary file 1 [file giag073_authors_response_to_reviewer_comments_original_submission.pdf]

Author's Response To Reviewer Comments

Please find below a point by point response to the reviewers.

Reviewer #1: ChatMDV captures the forefront of the intersection between artificial intelligence and bioinformatics. The technical pathway of integrating the LLM-RAG architecture with the MDV visualization platform is forward-looking. This work identifies and attempts to address the long-standing "technical gap" problem in single-cell data analysis. From an engineering implementation perspective, the authors have fully considered reproducibility and FAIR principles in the system design, constructing a complete closed loop from natural language understanding to code generation to interactive visualization. Its modular architecture also lays a good foundation for future expansion to multi-omics data types. However, as an engineering work and paper, there are still several issues that need to be addressed:

1. Regarding the evaluation of other tools, the commentary should be more precise and avoid subjective judgments. For example, in the discussion of MDV, "unclear where to begin the exploration" lacks specificity. Additionally, tools such as FlowAgent and CellAgent have already implemented LLM-driven automation of single-cell data analysis. Although they differ, they are more appropriate for comparison. The distinction from OMEGA should also be compared in greater detail. The paper states "To our knowledge, ChatMDV is the only platform that enables users to interactively visualise and further interrogate their transcriptomics data using natural language within a unified analytical interface." In the competitive AI-driven bioinformatics field, such claims should be avoided rather than limited by the subjective qualifier "To our knowledge."

We thank the reviewer for this important suggestion. We have improved the original phrasing to make it less subjective.
We have removed the sentence “To our knowledge, ChatMDV is the only platform…” from the Introduction.
We have revised the description of MDV limitations to provide more specific and objective wording (Background section, paragraph discussing MDV usability).e.g. Replacing ‘intuitive point and click graphical user interface’ with ‘graphical interface designed to support interactive data exploration’
We have added a new comparison paragraph (end of Background section) that explicitly contrasts ChatMDV with FlowAgent, CellAgent, and OMEGA in terms of deployment, interaction modality, and visualization capabilities.We have also ensured that comparisons are framed in terms of design differences rather than superiority claims, to avoid subjective interpretation.
These changes aim to provide a more balanced and precise positioning of ChatMDV within the existing landscape.

2. Different modules and steps should have corresponding evaluations. Evaluation and scoring should not only target final results; test sets should be established for intermediate steps, such as intent recognition and accuracy of generated code. For the 1-7 scoring of prompts, the difficulty of different tasks may vary and should have different weights.
We thank the reviewer for this insightful comment regarding evaluation granularity and prompt scoring. We agree that understanding the contribution of different components in a multi-stage system is important. However, ChatMDV is designed as an integrated pipeline in which intent interpretation, retrieval, and code generation are tightly coupled. For this reason, fully isolating these modules would not reflect realistic system behaviour, where correctness is ultimately determined by the final generated visualisation. To address this concern, we have strengthened the revised manuscript in two ways. First, we now include factor level diagnostic analyses based on the intermediate outputs already captured by our evaluation pipeline. Specifically, Supplementary Figure S1 shows the change in mean manual rating when individual sources of difficulty, such as omission of graph type, filtering requirements, parameter mapping, or underspecified biological context, are present versus absent. Complementary subgroup statistics are reported in Supplementary Table S4. Together, these analyses show that different aspects of prompt complexity have distinct and dataset-dependent effects on performance, with ambiguity-related factors

having the largest negative impact, especially in the more complex lung and TAURUS datasets.
Second, we now distinguish between multiple evaluation outcomes rather than relying on a single broad success metric. The revised manuscript reports:
Execution success (Ratings 2–5), reflecting whether code was generated and executed without pipeline failure.
Semantic success (Ratings 4–5), reflecting whether the output correctly addressed the user's intent.
Perfect success (Rating 5), reflecting fully correct outputs.
Although these analyses do not constitute fully independent module-level benchmarks, they provide interpretable diagnostics in the context of the pipeline design. For example, ambiguity in parameter specification or identifier mapping is most likely to affect the Data and Chart Planner Agent, whereas execution success more directly reflects the robustness of the Code Generation Chain. We make clear in the manuscript that these are indirect interpretations and not isolated module tests.
Regarding the 1–7 prompt complexity score, we agree that different criteria may contribute unequally to task difficulty. We therefore clarify that this score is intended as a descriptive additive index, not a weighted predictive model. Given the current evaluation set size, introducing fitted weights would risk overfitting and reduce interpretability. Instead, we report per-factor performance breakdowns (Supplementary Table S4) so that the effect of each criterion can be examined directly.

Overall, we believe these revisions improve the transparency and rigor of the evaluation while preserving the end-to-end assessment that is most relevant to practical use of ChatMDV.
3. The definition of the success rate metric is rather ambiguous. "Success rate" is defined as avoiding Rating 1 (complete failure), while Ratings 2-4 are all considered successful. Table 3 shows that the lung dataset has a significant proportion of Rating 3-4 (partial success), yet does not report the statistical significance of the exact match rate (Rating 5). Furthermore, outputs receiving a score of 1 have a complexity of only ≥2, which sounds too low. According to the authors' complexity criteria, user queries would typically satisfy multiple conditions simultaneously.
We thank the reviewer for highlighting the importance of appropriate control baselines and model transparency.

In the revised manuscript, we have expanded the evaluation to include a comparison across multiple LLM backends (GPT-4o, GPT-4o-mini, GPT-4.1, and GPT-5-mini). For each model, we report:
Execution success (Ratings 2–5): pipeline completes and produces a view
Semantic success (Ratings 4–5): output correctly addresses the user's intent but may require user to make minor changes in the GUI e.g. change axis height, label orientation
Perfect success (Rating 5): exact-match outputs where all required charts are correctly generated
This provides a controlled assessment of how model capability influences performance within the ChatMDV pipeline.
Across all datasets, we observe consistent trends:
Lower-capability models (e.g., GPT-4o-mini) show substantially reduced semantic and perfect success rates (e.g., 7–27% perfect success across datasets)
Higher-capability models (e.g., GPT-4.1 and GPT-5-mini) achieve significantly improved performance (up to 97% perfect success on PBMC3K)
These results demonstrate that model capability is a critical factor in achieving reliable natural language-driven visualisation.
We have updated the manuscript to explicitly state the model variants used (GPT-4o, GPT-4o-mini, GPT-4.1, GPT-5-mini) and report their comparative performance in the Results section. This ensures transparency and reproducibility.

We agree that an ablation study comparing direct LLM generation versus RAG-enhanced generation would be informative in principle. However, in the context of ChatMDV, such a comparison is not directly meaningful.
ChatMDV relies on a specialised MDV Python API that is not part of the standard training distribution of general-purpose LLMs. The RAG module provides essential grounding by retrieving API-aligned code templates, which guide the model toward generating syntactically valid and executable scripts. Without this grounding, a direct LLM baseline would typically fail to produce valid MDV-compatible code, resulting in near-zero execution success.

Therefore, rather than representing an incremental improvement, the RAG component is a necessary architectural element for enabling the task itself. For this reason, we focus on evaluating the integrated system and its dependence on model capability.

The multi-model comparison further shows that both retrieval-augmented prompting and sufficient model capability are required for high semantic correctness. We also note while higher-capability models (e.g., GPT-5-mini) improve performance, they incur increased computational cost and latency due to more extensive reasoning steps within the agent loop. This trade-off is now discussed in the revised manuscript. Overall, these additions provide a clearer and more controlled evaluation of the system, demonstrating how both model selection and retrieval grounding contribute to ChatMDV's performance.

5. The paper acknowledges that the lung dataset (~22GB) fails in typical desktop environments (8-12GB RAM), but does not clearly define the failure modes. It is recommended to supplement with tests of the maximum processable data scale under different memory configurations (16/32/64GB).

We have revised the manuscript to clarify that the observed failures on the lung dataset (~22GB when fully materialised in memory) are not caused by the MDV platform or the ChatMDV interface itself, but arise from memory-intensive downstream analytical operations (e.g. Scanpy-based computations) that require loading large data matrices into RAM.
ChatMDV, through MDV, is designed to be memory-efficient and scalable, using lazy loading and streaming from underlying datastores. As a result, interactive visualisation and querying remain functional even on modest hardware configurations, without requiring full dataset materialisation in memory.

To address this point, we have explicitly clarified in the manuscript the distinction between:
MDV-based visualisation, which is scalable and does not require full in-memory loading.
Downstream analytical steps (e.g., Scanpy), which may require substantial RAM depending on the operation.
This distinction explains why visualisation tasks succeed on large datasets, while certain analytical queries may fail under limited memory conditions.

We agree that profiling performance across different memory configurations (e.g., 16/32/64/128 GB) would be informative. However, such benchmarks would primarily reflect the behaviour of the underlying analysis libraries (e.g., Scanpy) and data representations, rather than the scalability of ChatMDV itself.
For this reason, we focus our evaluation on end-to-end system behaviour and clarify the expected hardware requirements for different classes of tasks. We now explicitly note that:
Visualisation workflows are lightweight and can run on standard desktop environments
Memory-intensive analyses benefit from higher-memory systems
We have updated the Methods and Discussion sections to:
Clarify the source of observed failures
Describe ChatMDV's memory-efficient architecture (lazy loading and streaming)
Highlight deployment recommendations for large-scale analyses
7. Do users have a way to verify that the final produced results match their intended data? A validation module is needed. If such validation methods already exist, please specify them in the paper. These are important as they relate to whether the user's cost of learning and correcting the AI is higher or lower than the cost of using MDV directly.

Thank you for this important point and we agree this should be made more clear.
ChatMDV currently has multiple ways to verify the outputs match their data.
The chat window includes an expandable dataset column/type/range summary.
We display the Python code that was executed for each answer
The output is rendered as an MDV view that the user can manually expect and adjust via the point and click interface.
The chat window also includes the following information:
Explanation as to why the visualisation used is appropriate and what parameters were used.
What biological insights can be gained from this visualisation

What subsequent analysis tasks could be performed based on these results
In addition, to make verification explicit and reduce the burden of checking/correcting outputs, we have added a 'verification summary' that is generated alongside each ChatMDV-produced view (see Figure 2b). This summary is derived from executed project metadata and the saved view JSON, and reports:
A data preview panel showing the fields and data to be used in the query (truncated if very long).
The datasources present in the project and the primary datasource used in the generated view
The chart types created and the exact columns/parameters used by each chart
Whether gene expression parameters were used
Code-level filtering/subsetting (rows before/after filtering)
Whether interactive filtering is enabled (e.g., via selection dialog charts).
This provides a "validation module" allowing users to confirm that the view corresponds to their intent (e.g., correct variables and groupings) and the correct data subset, without needing to manually interpret the full generated script. We describe the verification summary explicitly in the revised manuscript and include an example of the generated provenance summary in Figure 2b.

Reviewer #2: This manuscript presents ChatMDV, a natural language-driven bioinformatics visualization platform that integrates large language models (LLMs) with the MDV. Through an agent + RAG + code-generation architecture, ChatMDV translates user prompts into executable, reproducible Python analysis code and interactive MDV visualizations, and the authors provide a systematic evaluation on multiple scRNA-seq datasets at scale, including PBMC3K, the Human Cell Atlas lung atlas, and the TAURUS longitudinal study dataset. Overall, the work is technically solid, with strong system engineering and a high completeness. Below are a few remarks that I hope the authors can clarify in a revision.

1. The reported ">95% success rate" uses an overly generous definition and likely overestimates usability. A central claim is that ChatMDV achieves a success rate exceeding 95% in visualizing the evaluated datasets. However, the manuscript defines success as any rating except 1, i.e. effectively rating 2 - "View present but error-filled". However, the rating rubric explicitly states that rating 2 includes cases where "charts were present but did not address the question or contained significant errors." Counting these cases as "success" is too loose in my opinion, especially when success is interpreted by readers as semantic correctness or user-ready performance. Therefore, I think it would be great to distinguish execution success (rating over 2) and semantic success (rating over 4 and 5). Without this, the abstract ">95% success" risks being misleading.

We thank the reviewer for this important observation and agree that the original definition of "success rate" was overly permissive and could be misinterpreted as reflecting semantic correctness.
We have revised the manuscript to address this issue by:
Removing the ">95% success rate" statement from the abstract and main text
Introducing three clearly defined evaluation measures:
Execution success (Ratings 2–5): pipeline completes and produces a view
Semantic success (Ratings 4–5): output correctly addresses the user's intent but may require user to make minor changes in the GUI e.g. change axis height, label orientation
Perfect success (Rating 5): exact-match outputs where all required charts are correctly generated
We now emphasise semantic success (Ratings 4–5) as the primary measure of usability and report perfect success (Rating 5) separately to provide a stricter benchmark of performance.
These revised metrics are presented in the Results section and Table 1, ensuring a clearer and more accurate interpretation of system performance.

2. Evaluation prompts may be too well-formed. The prompt set is carefully designed and scored with a bespoke complexity scheme, and the manuscript notes that prompts were developed with expert input, literature review, and assistance from ChatGPT. While this is reasonable, it raises a concern that the final benchmark may be closer to "experienced user requests" than what many wet-lab or novice computational users would actually type, for example ambiguous phrasing, missing chart specifications, typos, etc. Therefore, adding a small additional benchmark of "inexperienced biologist prompts" would substantially

strengthen claims about democratizing analysis.
We thank the reviewer for this important suggestion. While our original benchmark focused on well-formed, expert-informed prompts, we conducted a small-scale exploratory evaluation using prompts provided by novice users (n=3) to better understand real-world usage patterns.
These prompts differed from the curated benchmark, often being underspecified or reflecting analysis oriented requests rather than direct visualisation instructions (e.g., “give me the top 5 marker genes in each cluster”). In such cases, ChatMDV previously attempted to generate visual outputs even when a textual summary would have been more appropriate, sometimes resulting in empty or uninformative views unless the user explicitly requested a text-based output (e.g., “write in a text box”).
Based on this observation, we have updated the system to support direct rendering of textual responses within the ChatMDV interface, allowing analytical outputs (e.g., lists of marker genes) to be presented without requiring explicit instruction from the user. This improves robustness to novice-style prompts and better aligns system behaviour with user intent.
We have updated the revised manuscript Results section as a qualitative evaluation of novice prompt behaviour and clarified that ChatMDV is designed to reduce, rather than eliminate, the need for structured query formulation.

3. Handling biological hallucinations is described at an engineering level as the manuscript acknowledges key failure modes and describes retries/error handling. However, these are mostly presented as engineering notes rather than as actionable, interpretable examples for end-users and reviewers. I think it would be useful to provide one or two concrete failure case analyses so that users can understand what ChatMDV can and cannot reliably do and how to proceed when it fails.
We thank the reviewer for this helpful suggestion. We agree that concrete, interpretable failure examples make the limitations more useful to readers. In the revised manuscript, we have expanded the ‘Limitations’ section to include specific examples of failure modes that users may encounter. In particular, we now describe cases where a user requests a gene by symbol while the dataset is indexed by Ensembl gene ID, which can lead to empty or incorrect outputs if the identifier mapping is not resolved. We also describe failures caused by ambiguous or poorly curated column names, which can result in incorrect field selection or failure to generate the intended visualisation. We further clarify that these issues are largely driven by dataset metadata quality rather than the visualisation framework itself, and note that they can often be mitigated through clearer field naming, better annotation, and explicit identifier mappings.

4. The word "democratizing" sounds quite strong as no one would describe the current bioinformatics practice as "autocratic". A more technical but accurate phrasing, like "reducing technical barriers" would be appreciated.
In this context, democratization is the process of making data more accessible to a wider range of people within an organization or society.
We thank the reviewer for this thoughtful suggestion and agree that the term “democratising” may be interpreted as overly strong or ambiguous in this context.
To improve clarity and maintain a more precise technical tone, we have revised the manuscript to replace “democratizing” with “reducing technical barriers” These changes better reflect the intention to enable a broader range of users to interact with complex biological datasets.

Cookie Preference Center

We use cookies which are necessary to make our site work. We may also use additional cookies to analyze, improve and personalize our content and your digital experience. For more information, see our Cookie Policy.

You may choose not to allow some types of cookies. However, blocking some types may impact your experience of our site and the services we are able to offer. See the different category headings below to find out more or change your settings.

Allow all

Manage Consent Preferences

Strictly Necessary Cookies

Always active

These cookies are necessary for the website to function and cannot be switched off in our systems. They are usually only set in response to actions made by you which amount to a request for services, such as setting your privacy preferences, logging in or filling in forms. You can set your browser to block or alert you about these cookies, but some parts of the site will not then work.

Functional Cookies

These cookies enable the website to provide enhanced functionality and personalisation. They may be set by us or by third party providers whose services we have added to our pages. If you do not allow these cookies then some or all of these services may not function properly.

Performance Cookies

These cookies allow us to count visits and traffic sources so we can measure and improve the performance of our site. They help us to know which pages are the most and least popular and see how visitors move around the site.

Targeting Cookies

These cookies may be set through our site by our advertising partners. They may be used by those companies to build a profile of your interests and show you relevant adverts on other sites. If you do not allow these cookies, you will experience less targeted advertising.

Cookie List

Clear

Apply Cancel

Consent Leg.Interest

Confirm my choices

Powe
