# Supplementary material for: ChatMDV: reducing technical barriers in bioinformatics analysis using large language models
Source: Gigascience. 2026 Jun 19;15:giag073. doi: 10.1093/gigascience/giag073 (PMC13452166; doi:10.1093/gigascience/giag073)
Supplement: Supplementary file 4 [file giag073_reviewer_1_report_original_submission.pdf]

Reviewer Report

Title: ChatMDV: Reducing Technical Barriers Using Large Language Models

Version: Original Submission **Date:** 12/17/2025

Reviewer name: Reviewer 1

Reviewer Comments to Author:

ChatMDV captures the forefront of the intersection between artificial intelligence and bioinformatics. The technical pathway of integrating the LLM-RAG architecture with the MDV visualization platform is forward-looking. This work identifies and attempts to address the long-standing "technical gap" problem in single-cell data analysis. From an engineering implementation perspective, the authors have fully considered reproducibility and FAIR principles in the system design, constructing a complete closed loop from natural language understanding to code generation to interactive visualization. Its modular architecture also lays a good foundation for future expansion to multi-omics data types. However, as an engineering work and paper, there are still several issues that need to be addressed:

1. Regarding the evaluation of other tools, the commentary should be more precise and avoid subjective judgments. For example, in the discussion of MDV, "unclear where to begin the exploration" lacks specificity. Additionally, tools such as FlowAgent and CellAgent have already implemented LLM-driven automation of single-cell data analysis. Although they differ, they are more appropriate for comparison. The distinction from OMEGA should also be compared in greater detail. The paper states "To our knowledge, ChatMDV is the only platform that enables users to interactively visualise and further interrogate their transcriptomics data using natural language within a unified analytical interface." In the competitive AI-driven bioinformatics field, such claims should be avoided rather than limited by the subjective qualifier "To our knowledge."
2. Different modules and steps should have corresponding evaluations. Evaluation and scoring should not only target final results; test sets should be established for intermediate steps, such as intent recognition and accuracy of generated code. For the 1-7 scoring of prompts, the difficulty of different tasks may vary and should have different weights.
3. The definition of the success rate metric is rather ambiguous. "Success rate" is defined as avoiding Rating 1 (complete failure), while Ratings 2-4 are all considered successful. Table 3 shows that the lung dataset has a significant proportion of Rating 3-4 (partial success), yet does not report the statistical significance of the exact match rate (Rating 5). Furthermore, outputs receiving a score of 1 have a complexity of only â‰¥2, which sounds too low. According to the authors' complexity criteria, user queries would typically satisfy multiple conditions simultaneously.
4. There is a lack of control baselines. The evaluation only tests ChatMDV's own success rate without establishing controls. For example, direct LLM generation without RAG: to compare whether RAG truly improves accuracy (an ablation study could be set up: GPT-4 direct vs RAG-enhanced). The paper also does not mention which version of GPT was tested or the differences arising from using various model versions.
5. The paper acknowledges that the lung dataset (~22GB) fails in typical desktop environments (8-12GB

RAM), but does not clearly define the failure modes. It is recommended to supplement with tests of the maximum processable data scale under different memory configurations (16/32/64GB).
7. Do users have a way to verify that the final produced results match their intended data? A validation module is needed. If such validation methods already exist, please specify them in the paper. These are important as they relate to whether the user's cost of learning and correcting the AI is higher or lower than the cost of using MDV directly.

Methods

Are the methods appropriate to the aims of the study, are they well described, and are necessary controls included? Choose an item.

Conclusions

Are the conclusions adequately supported by the data shown? Choose an item.

Reporting Standards

Does the manuscript adhere to the journal's guidelines on [minimum standards of reporting?](#) Choose an item.

Choose an item.

Statistics

Are you able to assess all statistics in the manuscript, including the appropriateness of statistical tests used? Choose an item.

Quality of Written English

Please indicate the quality of language in the manuscript: Choose an item.

Declaration of Competing Interests

Please complete a declaration of competing interests, considering the following questions:

- Have you in the past five years received reimbursements, fees, funding, or salary from an organisation that may in any way gain or lose financially from the publication of this manuscript, either now or in the future?
- Do you hold any stocks or shares in an organisation that may in any way gain or lose financially from the publication of this manuscript, either now or in the future?
- Do you hold or are you currently applying for any patents relating to the content of the manuscript?
- Have you received reimbursements, fees, funding, or salary from an organization that holds or has applied for patents relating to the content of the manuscript?

- Do you have any other financial competing interests?
- Do you have any non-financial competing interests in relation to this paper?

If you can answer no to all of the above, write 'I declare that I have no competing interests' below. If your reply is yes to any, please give details below.

I declare that I have no competing interests.

I agree to the open peer review policy of the journal. I understand that my name will be included on my report to the authors and, if the manuscript is accepted for publication, my named report including any attachments I upload will be posted on the website along with the authors' responses. I agree for my report to be made available under an Open Access Creative Commons CC-BY license (http://creativecommons.org/licenses/by/4.0/). I understand that any comments which I do not wish to be included in my named report can be included as confidential comments to the editors, which will not be published.

Choose an item.

To further support our reviewers, we have joined with Publons, where you can gain additional credit to further highlight your hard work (see: https://publons.com/journal/530/gigascience). On publication of this paper, your review will be automatically added to Publons, you can then choose whether or not to claim your Publons credit. I understand this statement.

Yes Choose an item.
