# Supplementary material for: ChatMDV: reducing technical barriers in bioinformatics analysis using large language models
Source: Gigascience. 2026 Jun 19;15:giag073. doi: 10.1093/gigascience/giag073 (PMC13452166; doi:10.1093/gigascience/giag073)
Supplement: Supplementary file 6 [file giag073_reviewer_2_report_original_submission.pdf]

Reviewer Report

Title: ChatMDV: Reducing Technical Barriers Using Large Language Models

Version: Original Submission **Date:** 1/14/2026

Reviewer name: Reviewer 2

Reviewer Comments to Author:

This manuscript presents ChatMDV, a natural language-driven bioinformatics visualization platform that integrates large language models (LLMs) with the MDV. Through an agent + RAG + code-generation architecture, ChatMDV translates user prompts into executable, reproducible Python analysis code and interactive MDV visualizations, and the authors provide a systematic evaluation on multiple scRNA-seq datasets at scale, including PBMC3K, the Human Cell Atlas lung atlas, and the TAURUS longitudinal study dataset. Overall, the work is technically solid, with strong system engineering and a high completeness. Below are a few remarks that I hope the authors can clarify in a revision.

1. The reported ">95% success rate" uses an overly generous definition and likely overestimates usability. A central claim is that ChatMDV achieves a success rate exceeding 95% in visualizing the evaluated datasets. However, the manuscript defines success as any rating except 1, i.e. effectively rating 2 - "View present but error-filled". However, the rating rubric explicitly states that rating 2 includes cases where "charts were present but did not address the question or contained significant errors." Counting these cases as "success" is too loose in my opinion, especially when success is interpreted by readers as semantic correctness or user-ready performance. Therefore, I think it would be great to distinguish execution success (rating over 2) and semantic success (rating over 4 and 5). Without this, the abstract ">95% success" risks being misleading.
2. Evaluation prompts may be too well-formed. The prompt set is carefully designed and scored with a bespoke complexity scheme, and the manuscript notes that prompts were developed with expert input, literature review, and assistance from ChatGPT. While this is reasonable, it raises a concern that the final benchmark may be closer to "experienced user requests" than what many wet-lab or novice computational users would actually type, for example ambiguous phrasing, missing chart specifications, typos, etc. Therefore, adding a small additional benchmark of "inexperienced biologist prompts" would substantially strengthen claims about democratizing analysis.
3. Handling biological hallucinations is described at an engineering level as the manuscript acknowledges key failure modes and describes retries/error handling. However, these are mostly presented as engineering notes rather than as actionable, interpretable examples for end-users and reviewers. I think it would be useful to provide one or two concrete failure case analyses so that users can understand what ChatMDV can and cannot reliably do and how to proceed when it fails.
4. The word "democratizing" sounds quite strong as no one would describe the current bioinformatics practice as "autocratic". A more technical but accurate phrasing, like "reducing technical barriers" would be appreciated.

Methods

Are the methods appropriate to the aims of the study, are they well described, and are necessary controls included? Choose an item.

Conclusions

Are the conclusions adequately supported by the data shown? Choose an item.

Reporting Standards

Does the manuscript adhere to the journal's guidelines on minimum standards of reporting? Choose an item.

Choose an item.

Statistics

Are you able to assess all statistics in the manuscript, including the appropriateness of statistical tests used? Choose an item.

Quality of Written English

Please indicate the quality of language in the manuscript: Choose an item.

Declaration of Competing Interests

Please complete a declaration of competing interests, considering the following questions:

- Have you in the past five years received reimbursements, fees, funding, or salary from an organisation that may in any way gain or lose financially from the publication of this manuscript, either now or in the future?
- Do you hold any stocks or shares in an organisation that may in any way gain or lose financially from the publication of this manuscript, either now or in the future?
- Do you hold or are you currently applying for any patents relating to the content of the manuscript?
- Have you received reimbursements, fees, funding, or salary from an organization that holds or has applied for patents relating to the content of the manuscript?
- Do you have any other financial competing interests?
- Do you have any non-financial competing interests in relation to this paper?

If you can answer no to all of the above, write 'I declare that I have no competing interests' below. If your reply is yes to any, please give details below.

I declare that I have no competing interests.

I agree to the open peer review policy of the journal. I understand that my name will be included on my report to the authors and, if the manuscript is accepted for publication, my named report including any attachments I upload will be posted on the website along with the authors' responses. I agree for my report to be made available under an Open Access Creative Commons CC-BY license (http://creativecommons.org/licenses/by/4.0/). I understand that any comments which I do not wish to be included in my named report can be included as confidential comments to the editors, which will not be published.

Choose an item.

To further support our reviewers, we have joined with Publons, where you can gain additional credit to further highlight your hard work (see: https://publons.com/journal/530/gigascience). On publication of this paper, your review will be automatically added to Publons, you can then choose whether or not to claim your Publons credit. I understand this statement.

Yes Choose an item.
